# Development of a Mobile Phone App for Measuring Striking Response Time in Combat Sports: Cross-Sectional Validation Study

**DOI:** 10.2196/14641

**Published:** 2019-11-11

**Authors:** Victor Coswig, Jader Sant' Ana, Maicon Nascimento Coelho, Antonio Renato Pereira Moro, Fernando Diefenthaeler

**Affiliations:** 1 Universidade Federal do Pará Departamento de Educação Física Castanhal Brazil; 2 Universidade Federal de Santa Catarina Departamento de Educação Física Florianopolis Brazil

**Keywords:** reaction time, martial arts, mobile apps, software validation

## Abstract

**Background:**

TReaction is a mobile app developed to determine strike response time at low cost and with easy application in combat sports. However, the validity and accuracy of the response time obtained by the TReaction app has not yet been evaluated.

**Objective:**

This study aimed to test the validity and reliability of the TReaction app in measuring motor response time in combat sports.

**Methods:**

A total of two athletes performed 59 strikes to assess the response time upon visual stimulus using the TReaction app simultaneously with a high-speed camera. Accuracy of the measure was verified using a computer simulator programmed to discharge visual stimuli and obtain the response time. Pearson correlation, Student *t* test for dependent samples, and the Bland-Altman analysis were performed. Accuracy was verified using the intraclass correlation coefficient. Effect size (g) and the typical error of measurement (TEM) were calculated. The significance level was set at *P*<.05.

**Results:**

No significant difference (*P*=.56) was found between both systems. The methods presented a very strong correlation (*r*=0.993). The magnitude of differences was trivial (g<0.25), and TEM was 1.4%. These findings indicate a high accuracy between the computer screen and the mobile app measures to determine the beginning of the task and the response time.

**Conclusions:**

Our findings suggest that the TReaction app is a valid tool to evaluate the response time in combat sports athletes.

## Introduction

### Background

The success of combat sports is often determined by fast motor actions in response to a given stimulus [1-3]. Response time, defined as the time required to perform a voluntary movement after a stimulus [4,5], is an important variable that can be improved and monitored in combat sports conditioning [6-9]. A greater success during competition could be associated with faster response time value [10,11].

### Technology Systems to Measure Response Time

To measure response time from a given visual or audible stimulus, different systems have been proposed to determine the time to perform a specific task or motor action. Most systems use cameras with high sampling frequency [12,13] or accelerometers [14,15] to obtain spatial and temporal information of a determined motor gesture or task. Moreover, some systems measure response time based on visual stimuli shown on a computer screen in time intervals as previously defined. In these cases, response time to visual stimulus is measured from the moment that the user presses the computer keyboard [16,17].

### Limitations of Systems to Measure Response Time

Despite the quick results provided by those systems in which computer keyboards are used, they lack specificity (specifically motor gestures) and ecological validity, as combat sports actions are not performed during the task. In addition, systems involving kinematics (motion analysis) or accelerometry to measure response time usually require more time to prepare, collect, and analyze the data. Moreover, these systems are expensive and often have low applicability to athlete training routines.

### Objective and Research Questions

To minimize the aforementioned limitations, the use of a specific mobile phone app (TReaction app, ETS4ME, São José, SC, Brazil) has been proposed to determine the response time of different motor actions (strikes) with a low-cost, highly applicable, and ecologically valid solution. The TReaction app measures the motor response time between the flash fire and sound waves produced from contact with the target through the mobile phone microphone. However, as far as we know, the response time measurement validity obtained by the TReaction app has not been tested. In fact, the use of mobile apps has been growing exponentially in fitness, health, and sports science, which raises the need for scientific evidence [18]. Therefore, this study aimed to test the validity and reliability of the TReaction app in measuring motor response time in combat sports.

## Methods

### Experimental Design

Data collection for this study was performed in 2 sessions. In the first session, a response time test was performed using the TReaction app simultaneously with a high-speed camera (visual system). In the second session, the TReaction app measurement reliability was validated with a repeated measures design using a simulator. This simulator was programmed to verify the accuracy of the visual stimuli and the response time with previously programmed fixed intervals.

### Subjects

The 2 subjects of this study were selected intentionally. These individuals were male black belt athletes with significant experience in regional and national taekwondo competitions. Furthermore, each selected subject had to have at least 10 years of experience in a modality of combat sport. Before participating in the study, all participants were informed and provided written consent about the specific aims and methods of the study along with possible study risks. The local research ethics committee approved this study (protocol number 145.882). All procedures were performed in accordance with the Declaration of Helsinki.

### Procedures

#### Motor Response Time

The TReaction app was used to determine the motor response time and the accuracy of the measurement was validated through a repeated measures design that examined motor gesture accuracy during contact with an impact pad. The measurements of values obtained by the mobile app (TReaction app) were compared with those obtained using a digital high-speed camera, which is considered the gold standard method.

#### Mobile App

**Figure 1:**
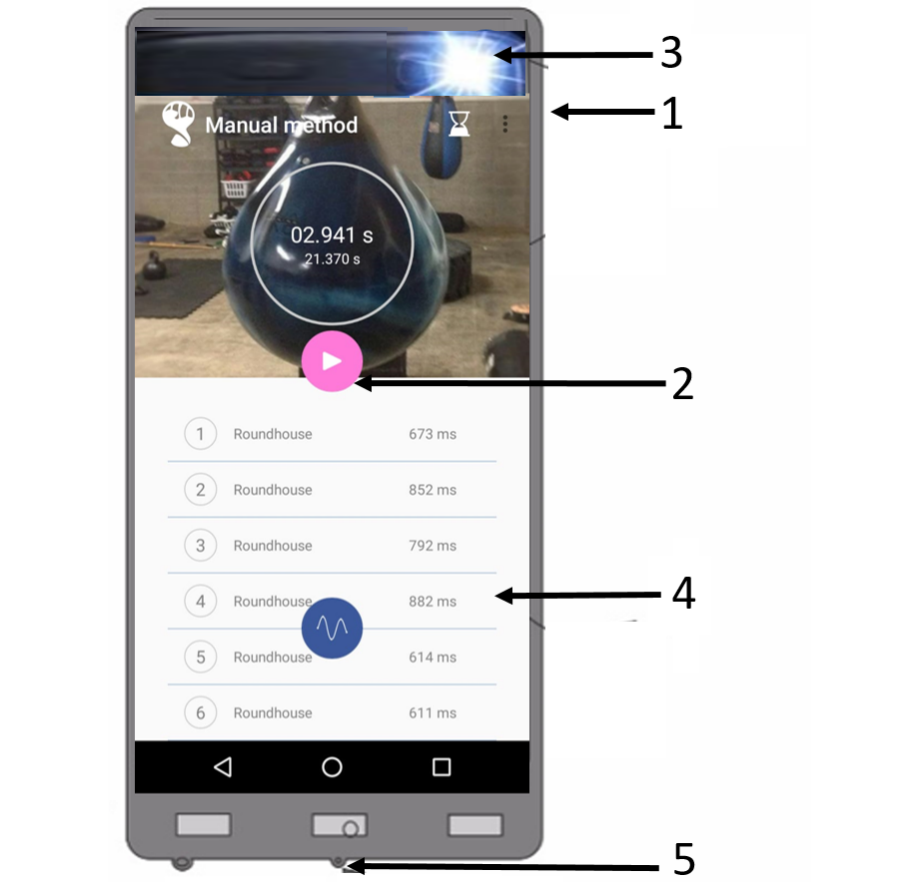
Representation of the TReaction app system. See text above for explanation of numbered points.

The response time obtained from the TReaction app is provided as shown in Figure 1 and described below:

- Number 1 represents the mobile phone;
- Number 2 represents a play button via touchscreen technology present in the mobile app interface and that activates the mobile phone camera flash through an algorithm software developed in Java programming language using integrated development environment Android Studio (Apache Software Foundation, Maryland, EUA) and that allows to acquire and process data;
- Number 3 represents the mobile phone camera flash, emitting the visual stimulus and indicates the beginning of the response time to perform a gesture or task to reach a contact target (ie, reach an impact pad for kicks);
- Number 4 represents when contact with the target occurs and a sound wave is generated and processed through the software developed to accurately identify and point response time test results obtained;
- Number 5 represents the mobile microphone to audio capture.

#### High-Speed Camera

To accurately identify the strike response time (defined as the time interval between the light signal and the moment of contact with the target), we performed a simultaneous motion analysis using a high-speed digital camera (Fastec TS4, Motion Engineering Company) with a sampling frequency of 1000 Hz and compared it with repeated measures. The response time was determined using a software analysis (Fastec FasMotion, Motion Engineering Company), capturing the first frame when the flashlight appeared and the moment of the material restitution caused by the contact of the foot with the contact pad.

#### Motor Response Time Measurement Protocol

**Figure 2:**
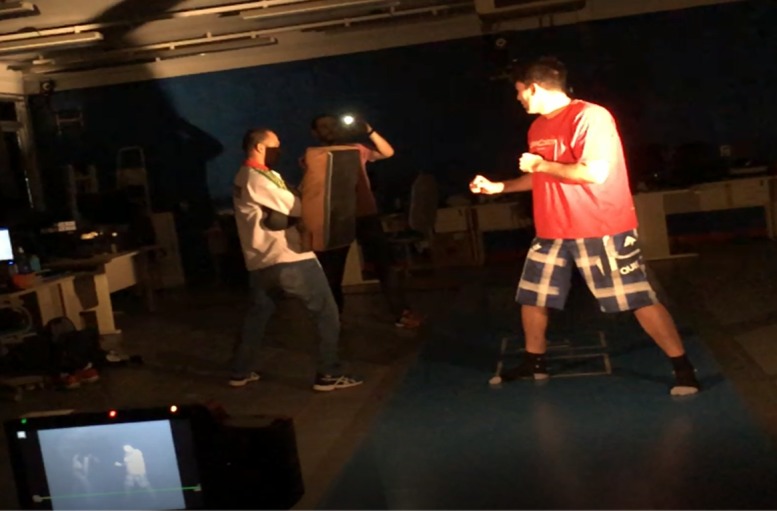
Set up of the protocol test position to identify response time of the strikes.

Both athletes performed a total of 59 strikes using the roundhouse kick technique at the height of the trunk. To calculate the response time, 2 different mobile phone devices were used (30 strikes registered with a Samsung Galaxy J7 Neo with Android 7.0 system, and 29 with a Motorola G Moto G Play with Android 6.0.1 system). The tests were conducted as shown in Figure 2, with strikes being performed aiming to reach the impact pad in response to the visual stimulus (camera flash signal). To perform the strike, the athlete positioned himself in front of the impact pad that was held by an experienced researcher. The mobile device with the TReaction app was held by another researcher and positioned sideways to the impact pad so that the camera flash was directed to the athlete’s vision field. The high-speed camera was positioned perpendicular to the task plane of motion and in such a way to include the athlete, the impact pad, and the mobile’s camera flash. Both athletes had previous experience with this protocol.

#### Accuracy Measurement Protocol

To verify the accuracy of the measurement over repeated measures, a software running with an algorithm developed in Java language, simulating the strike sound and simultaneous change in the computer monitor screen color (Notebook Acer aspire E 15, Acer), was used. The monitor screen was alternating between black and white with the following epochs previously fixed at 3014, 3004, 3014, and 3002 ms. This configuration was chosed to test the consistency of the changes in the monitor screen. First, the TReaction app was set up in the consecutive audio capture mode to verify response time reliability via audio acquisition. Then it was set up in the flash drive mode repeatedly for each auditory stimulus emitted by the computer, with following epochs previously fixed at 3014, 3004, 3014, and 3002 ms. At this moment, the high-speed camera was used to simultaneously record repeated responses of the mobile phone camera flash and the color change of the monitor screen.

### Statistical Analysis

Descriptive statistics (mean [SD]) were used to present the results, and normality was verified using the Shapiro-Wilk test. Pearson linear correlation was used to verify the relationship between response time obtained by the TReaction app and high-speed camera. A paired Student *t* test was used to test differences between both methods. The magnitude of the differences was verified from the effect size (*g*), and it was then scaled for trivial (0.25), small (0.25 to 0.50), moderate (0.50 to 1.0), or large (1.0) effects [19]. The typical error of measurement (TEM) was estimated by dividing the SD of the difference score by √2 [20]. The Bland and Altman [21] analyses were used to test agreement between the methods. The intraclass correlation coefficient (ICC) was established to verify the accuracy of repeated measures. Data processing and analysis were performed using Microsoft Office Excel 2007, SPSS 17.0 (IBM Corp) and GraphPad Prism 5.01. The statistical significance level was set at *P*<.05.

## Results

Table 1 shows the mean and SD values, level of significance of the differences, the magnitude of the effect size, and the TEM for the response time obtained by the high-speed camera and the TReaction app.

For mean response time values between the high-speed camera and the TReaction app, there was no difference, and the magnitude of the differences obtained was classified as trivial. The methods to determine the response time presented a strong correlation, and the TEM pointed to the confidence in the measurement obtained from the TReaction app in relation to the high-speed camera.

**Figure 3:**
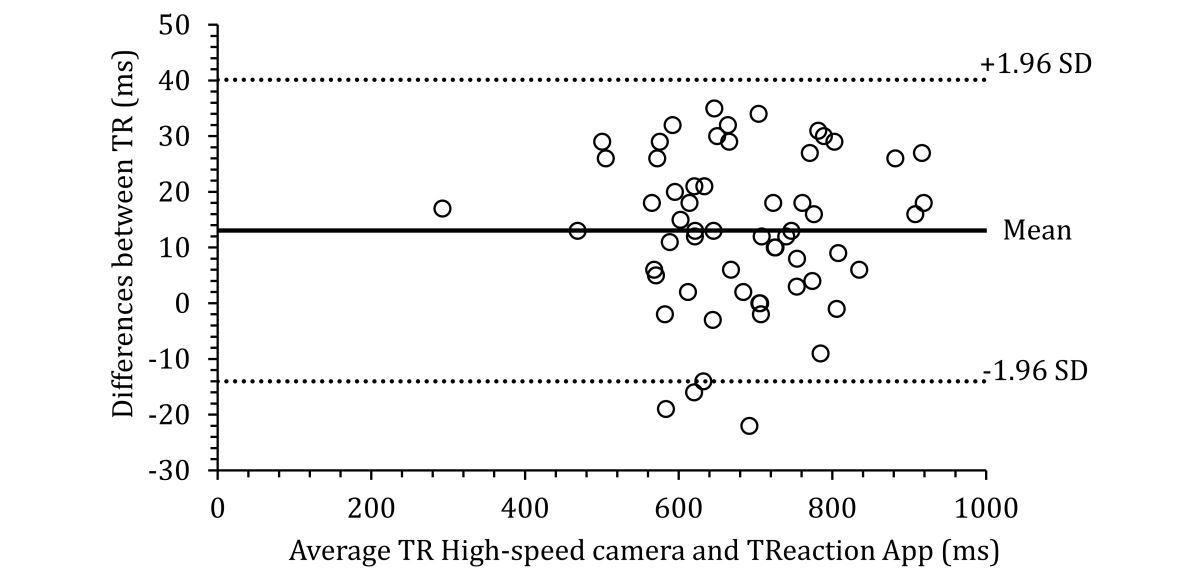
Bland and Altman plots describing the agreement of measurements of response time obtained with high speed camera and TReaction app.

The Bland and Altman plots describing the agreement of response time measurements obtained with a high-speed camera and the TReaction app is presented in Figure 3. Response time presented a bias of 13.05 ms. The 95% limits of agreement for differences of response time between the high-speed camera and TReaction app were 27.06 ms or −14.01 ms and 40.11 ms (below and above bias, respectively).

**Table 1:** Mean and standard deviation for the response time obtained using the high-speed camera and TReaction app (n=59).

Parameter	High-speed camera, mean (SD)	TReaction app, mean (SD)	*r*^a^ value	*P* value	g (ES^b^)^c^	TEM^d^ (%)
Response time (ms)	673 (115)	686 (116)	0.993	.56	0.113	9.2 (1.4)

^a^Pearson linear correlation.

^b^ES: effect size.

^c^Magnitude of the differences.

^d^TEM: typical error of measurement (absolute and relative values).

Table 2 shows the values of the repeated measures of audio emission time and computer screen color change and the capture of the audio system obtained with the TReaction app. There was no difference in mean time values in comparison with the values obtained by the repeated measures test for change in the computer screen color and to the response time obtained by the audio capture using the TReaction app. The magnitude of the differences obtained was classified as trivial and the TEM pointed to reliability showed a strong correlation established by the ICC for the repeated measures.

**Table 2:** Mean and standard deviation for the comparison of the repeated measures of the audio emission time and the screen change of the simulator, as well as the capture of the audio system obtained with the TReaction app (n=52).

Parameter	Audio and screen (computer), mean (SD)	Audio TReaction, mean (SD)	*P* value	*g* (ES^a^)^b^	TEM^c^ (%)	ICC^d^
Time (ms)	3009 (5.5)	3008 (5.6)	.37	0.011	0.04 (0.001)	0.998

^a^ES: effect size.

^b^Magnitude of the differences.

^c^TEM: typical error of measurement (absolute and relative values).

^d^ICC: intraclass correlation coefficient.

Table 3 shows repeated measurement values of the mobile phone camera flash opening time to trigger the motor response onset and the computer screen change time. There was no difference between mean values of the flash opening time and values obtained by the repeated measurements test using the TReaction app and the measures of the color change in the computer screen. The magnitude of the differences obtained was classified as trivial, and the TEM pointed to accuracy; a strong correlation was established between the repeated measurements to determine the visual stimulus to perform a motor response.

**Table 3:** Mean and standard deviation for the comparison between values of the flash opening time and values obtained by the repeated measurements test using the TReaction app and the measures of the color change in the computer screen (n=52).

Parameter	Flash (Fastec)	Screen (Fastec)	*r*^a^ value	*P* value	g (ES^b^)^c^	TEM^d^ (%)
Time (ms)	3007 (16.5)	3009 (5.5)	0.846	.43	0.103	0.8 (0.03)

^a^Pearson linear correlation.

^b^ES: effect size.

^b^Magnitude of the differences.

^c^TEM: typical error of measurement (absolute and relative values).

## Discussion

### Principal Findings

This study aimed to verify the validity of the TReaction app, which was designed to evaluate the motor response time of combat sports athletes. In addition, this study aimed to verify if the setup used by the TReaction app to determine the beginning of the task (flashlight stimulus) and the time of contact with the target (time to perform the task) would reach acceptable levels of accuracy. To the best of our knowledge, this is the first study to investigate the validity of the TReaction app. The main results showed no differences between the mean response time of all strikes evaluated using the TReaction app and those obtained by the high-speed camera, which is supported by the effect size observed (trivial). The response time results showed a strong correlation between both TReaction app and high-speed camera (gold standard) methods. Besides, the TEM in our study (9.2 ms) is lower than the value (30 ms) observed previously with single-beam photocells across short-sprint studies [22].

When using the TReaction app, considerations must be given to the values of differences by the inference and the degrees of agreement of the Bland and Altman plot. Our results show an average difference of 13 ms for response time between TReaction app and high-speed camera. Response time presented a bias of 13.05 ms, and the 95% limit of agreement for the differences between the high-speed camera and TReaction app was 27.06 ms with 3 individual samples slightly outside the limit of agreement. Although the response time observed in our study presented bias higher than the value (0.35 ms) detected by [23], the variance of the magnitude of different values can be considered similar to the values observed by the authors (95% limits of agreement −11.78 to 12.39 ms). In the above-mentioned study, the authors also observed 3 individual samples slightly outside the limit of agreement, and the difference between systems to measure response time during the task was higher than 17 ms for an average time of 246 ms.

This study demonstrated that the difference between the TReaction app and high-speed camera observed is associated with the distinct method that the TReaction app uses to calculate the time between the visual stimulus (flash fire) to perform the task and the moment to determine the motor response time. The TReaction app calculates response time based on the sound wave generated by the impact with the target object. This measure presented a high ICC and no differences between the audio output and the previously programmed computer software.

The kinematics is among the most accurate systems used to measure motor gestures associated with performance such as vertical jump [24,25], especially when the goal is to accurately measure the performance of motor gestures in combat sports [13,26]. However, high cost and low applicability of these kinematics systems make it challenging to control essential parameters in combat sports. Therefore, the cost-effectiveness and high applicability of utilizing the TReaction app make it a viable alternative for accurate response time measurements in combat sports.

### Study Limitations

One limitation of the TReaction app may be associated with the small variability observed in the firing time of the flash over repeated measures, which increases the probability of incorrectly identifying the moment of the initial stimulus to perform the task. However, in this study, a high-speed camera (1000 Hz) was used to establish the response time with maximum accuracy, and no differences were observed when compared with the response time values obtained by TReaction app. Another aspect to consider when interpreting our findings is regarding the limited sample size, as a higher variability can be expected from international and national level athletes (coefficient of variation 2.9% and 6.1%, respectively) in response time when repeated measures of kicks are performed [27], which means that our perspectives are limited for this group. Although we believe that it did not reduce the strength of our findings, future studies should be encouraged to investigate TReaction feasibility in different modalities and subjects’ characteristics, such as competitive levels.

### Final Considerations

Finally, the reliability observed for computer screen color change time, previously programmed to occur simultaneously to the auditory stimulus at predefined times, was also verified. Results observed allow the use of the screen color change as an alternative to be used as a visual stimulus to initiate a motor task. Therefore, we suggest that the TReaction app can accurately measure response time during different techniques used in combat sports. This is relevant because response time is an important variable for combat sports [8,10,15,26]. As the TReaction app is a viable alternative to obtain and control the response time for combat sports athletes, it can possibly be used by athletes in other sports, physical trainers, and coaches to obtain time interval measurements between stimulus onset and the response time required for contacting a target with a strike.

### Conclusions

The results of this study suggest that TReaction app is a valid and reliable tool for measuring response time in combat sports. However, the TEM and observed interference of agreement should be considered when comparing the response time values obtained by TReaction app with other kinematic measurements.

The TReaction app provides an easily executable test that provides relevant sport-specific data regarding motor response time. Outcomes from the TReaction app suggest that athletes and coaches involved in combat sports can obtain reliable continuous motivational feedback leading to accurate diagnostic and performance criteria. The TReaction app can achieve and better control training-induced adaptations as well as help choose interventions that will help achieve maximum combat sports success. In addition, the TReaction app showed to be a valid tool that is both cost-effective and highly applicable to combat sports practitioners.

## Abbreviations

ICC: intraclass correlation coefficient
TEM: typical error of measurement
